# Safety Distance Identification for Crane Drivers Based on Mask R-CNN

**DOI:** 10.3390/s19122789

**Published:** 2019-06-21

**Authors:** Zhen Yang, Yongbo Yuan, Mingyuan Zhang, Xuefeng Zhao, Yang Zhang, Boquan Tian

**Affiliations:** 1 Department of Construction Management, Dalian University of Technology, Dalian 116024, China; yangz@mail.dlut.edu.cn (Z.Y.); yongbo@dlut.edu.cn (Y.Y.); tian_boquan@mail.dlut.edu.cn (B.T.); 2 School of Civil Engineering, Dalian University of Technology, Dalian 116024, China; zhaoxf@dlut.edu.cn (X.Z.); zhangyang2015@mail.dlut.edu.cn (Y.Z.)

**Keywords:** construction management, construction safety, cranes, imaging techniques, safety distance

## Abstract

Tower cranes are the most commonly used large-scale equipment on construction site. Because workers can’t always pay attention to the environment at the top of the head, it is often difficult to avoid accidents when heavy objects fall. Therefore, safety construction accidents such as struck-by often occurs. In order to address crane issue, this research recorded video data by a tower crane camera, labeled the pictures, and operated image recognition with the MASK R-CNN method. Furthermore, The RGB color extraction was performed on the identified mask layer to obtain the pixel coordinates of workers and dangerous zone. At last, we used the pixel and actual distance conversion method to measure the safety distance. The contribution of this research to safety problem area is twofold: On one hand, without affecting the normal behavior of workers, an automatic collection, analysis, and early-warning system was established. On the other hand, the proposed automatic inspection system can help improve the safety operation of tower crane drivers.

## 1. Introduction 

Countries all over the world pay great attention to security issues due to the dynamic and complex work conditions of construction sites [1]. However, construction workers’ safety situation is grim, as the quantity of accidents is still rising recent years. In 2017, both the quantity of safety accidents and the quantity of deaths have increased compared with 2016. Several dynamic factors such as wires carrying the load are dynamically stressed, causing the lift is so difficult to perform. Data display how that lifting damages are the most common, accounting for 10.40% of all accidents [2]. There are two reasons for this result. On the one hand, too many projects under construction lead to the large amount of accidents. On the other hand, these accidents also reflect the problems of extensive safety management, inadequate safety protection, and weak safety awareness. Although some countries have developed effective programs to improve Occupational Safety & Health (OSH) [3], lifting accidents are a scourge worldwide. 

To deal with safety problems, many scholars explore the empirical factors influencing unsafe behaviors and accidents on construction sites [4,5]. By investigation, Tian Shuicheng finds that the occurrence of accidents requires the existence of dangerous sources, workers, and unsafe behaviors [6]. With this discovery, he establishes “three types hazard theory” to explain the importance of managing the relationship between on-site workers and hazards. Moreover, according to the accident pyramid, Heinrich (1959) proposes that for every 300 unsafe acts, there are 29 minor injuries and 1 major injury. In other words, it suggests the ratio between major injuries, minor injuries, and no-injury accidents is 1:29:300 [7]. This means that the farther away from the danger zone, the less safety incidents occur.

While the theory is improving, the auxiliary technology is gradually developing at the same time. At present, some supervision units at construction sites use manual methods for regular inspections. However, this kind of supervision has certain limitations that it is impossible to conduct supervision work. As a result, sensor technology begins to gradually emerge in the field of construction supervision. These technology include RFID (radio frequency identification) technology for material management [8], Smartphone-based worker efficiency management technology [9], location monitoring system based on ultrawide band technology, RF (radio frequency) remote sensing, WIFI (wireless fidelity) [10,11,12], and tracking workers’ paths relying on video streams [13,14]. Although each assistive technology has its own advantages, there is still room for improvement in the sensing technology.

**Figure 1:**
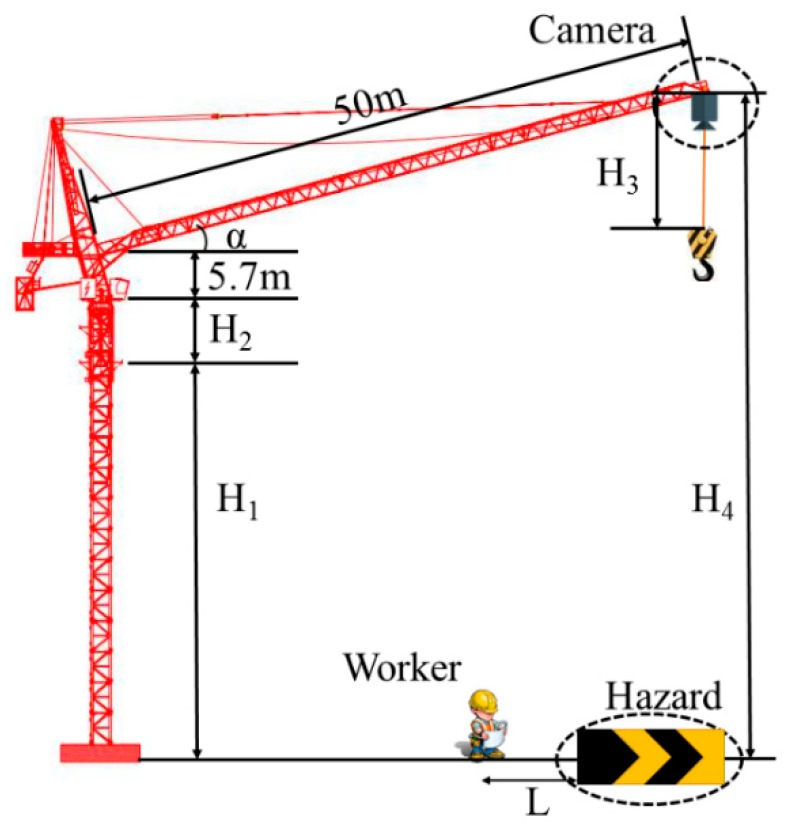
Tower crane camera installation diagram.

The aim of this paper is to strengthen the workers safety distance management by taking the high-altitude falling object as an example. As shown in Figure 1, it is assumed that hazard is a dangerous zone for falling cargo hanged by hooks. This paper proposes a novel safety distance monitoring method between hazard and workers, and core content consists of the following two parts: First, the tower crane camera videos are collected at construction site and image recognition work is performed on dangerous zone, workers and tower crane hooks by the MASK R-CNN method. Second, we extract the pixel coordinates of the “Mask“ layer, and calculated the safety distance by pixel and actual distance conversion method. 

## 2. Literature Review 

Image recognition refers to the technique of using a computer to process, analyze, and understand images to identify targets and objects in various modes.

### 2.1. Image Recognition Technology

In 1980, the neocognitron decomposed a visual pattern into a number of sub-patterns and then proceeded into a hierarchically-level connected feature plane for processing [15]. This marked the birth of the first initial convolutional neural network. At the same time, it was the first application of the Receptive Wild concept in the field of artificial neural networks. However, CNN method still had two shortcomings at that time: (1) The traditional CNN method required a fixed-size input image and the normalization method led a object image to be cropped or warped, which caused the information of the input CNN to be lost; (2) Since each Proposal Region need to entered the CNN network calculation, and there were a large number of overlaping by thousands of Regions, so repeated feature extraction brought huge computational waste. To solve the problems, Behnke proposed a neural abstraction pyramid method [16]. The feedforward architecture of the convolutional neural network could be extended by horizontal and feedback connections in the neural abstraction pyramid and the resulting recurring convolutional network allows for flexible incorporation of context information to iteratively resolve local ambiguities. Contrary to previous models, CNN produced the highest resolution image output, which solved the aforementioned problems. Dolan implemented CNN through GPU [17], marking a more efficient way to implement CNN and it stood out in the 2012 ImageNet contest. Based on CNN, Girshick proposed Fast R-CNN method [18], which accelerated RCNN by the following three improvements: 1) The simplified version of the ROI pooling layer and the added candidate box mapping function enabled the network to back propagate, then it solved the overall network training problem of the SPP (Spatial Pyramid Pooling); 2) Multitasking Loss layer; 3)Fully connected layer was accelerated by SVD (Singular Value Decomposition). However, Fast-R-CNN was affected by “proposal extraction” and it taked about 2 s for all Proposals to extract an image. To solve this, Ren Shaoqing proposed a new Faster R-CNN algorithm [19], which introduced a full convolutional neural network-RPN (Region Proposal Network). Moreover, the RPN only taked 10 m s to extract a proposal by sharing the convolutional layer feature.

### 2.2. Computer Vision Applications in Construction

As the accuracy and timeliness of image recognition have been greatly developed, many scholars applied it to on-site safety inspection and supervision directly [20,21,22]. Generally, there are two types of applications that are directly identified. The first category directly identifies the wearable devices that protect workers, such as, to reduce head and neck injuries, Fang Qi trained 100,000 images in various environments through Fast R-CNN, and the generated model can accurately identify the hardhats [23]. Fang Weili used computer vision-based approach for safety harness and equipment detection to prevent workers from danger [24,25]. Another category directly identifies the on-site equipment, for example, Zdenek Kolar developed a safety guardrail detection model based on convolutional neural network to identify the unsafe conditions on site [26]. In 2015, Li Feifei proposed a model that generate natural language descriptions of image regions based on weak labels [27]. Immediately after that, semantic recognition technology gradually matured, as it can extract more valuable information from the image. Ding Lieyun et al. developed a hybrid model CNN+LSTM (long short-term memory) to analyze the workers’ unsafe actions [28]. Similarly, Luo Hanbin improved convolutional neural network (CNN) that integrates Red–Green–Blue (RGB), optical flow, and gray stream CNNs, and the improved CNN could detect the worker’s activity to assist the project for personnel management [29]. Moreover, Dominic Roberts combined Unmanned Aerial Vehicle photography and image recognition technology to track on-site cranes and estimate 3D crane pose [30]. All of above-mentioned studies have provided more advanced technology for on-site safety management.

**Figure 2:**
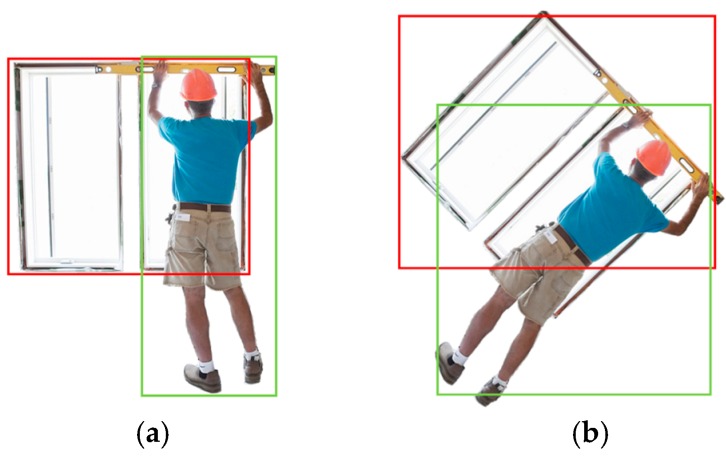
Comparison of recognition results before (**a**) and after (**b**) camera reversal.

Accompanied by various advantages, Fast R-CNN technique also has a common problem in practical applications. As shown in Figure 2a, the green and red boxes represent the candidate boxes of workers and windows, respectively. The spatial interaction of two candidate boxes used to describe the positional relationship between workers and windows, and to analyze whether workers are at risk of falling [31]. However, the method is limited by the mechanism of image recognition: the four sides of the candidate box are always horizontal and vertical. As shown in Figure 2b, once the camera is tilted, the candidate box will deviate from the captured object, this will cause a large error in the results.

### 2.3. Safety Distance in Construction

Laws and regulations have been enacted to prevent construction accidents. Chi and Caldas [32] established safety rules based on three fundamental risk factors regarding earthmoving and surface-mining activities: (1) excessive operation speeds, (2) dangerous access to unsafe areas, and (3) close proximity between objects, such as heavy machinery and workers. Some excerpts on the safety distance [33] are shown in Table 1:

Based on a predefined maximum allowable exposure to the safety risk, Tsah identified a statistical zones [34]. The resultant limits of the zones are specified in Table 2, which is a method to control the movement of workers on the site, and to proactively impose certain limits to their hazard exposure.

Although the requirements for safety distance in the specifications are very detailed, due to the actual operability is weak and the safety management is rough, resulting in the negligence or misjudgement of participants in tower crane operations. Therefore, the overall aim of this research is to propose a maneuverable, high-precision auxiliary management technology based on the Mask R-CNN method for the crane drivers.

## 3. Method

**Figure 3:**
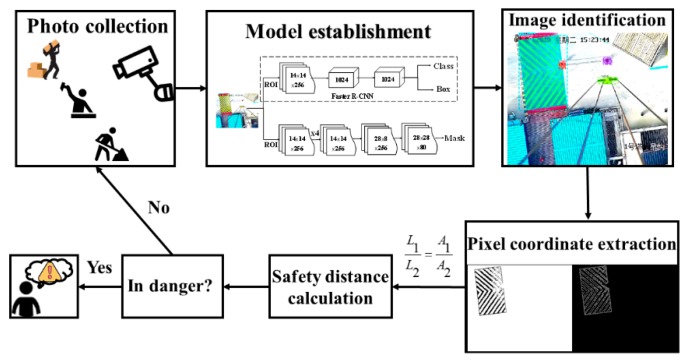
Overall framework of the proposed method.

This paper presents an automated method to inspect the distance between builder and dangerous edge. Figure 3 illustrates the main operation steps.

### 3.1. Collection of Training Pictures

The image resolution determines the quality of the image recognition model. Now, there are many platforms for image crowdsourcing, but no professional platform for building construction services. In order to collect photos and build a complete on-site hazard identification model, an image crowdsourcing platform has been established. All platform users can create their own account, upload, mark, describe the on-site photos, and choose whether to public these photos or not. Moreover, we created a WeChat applet “plate platform” to facilitate the workers collect and upload images through mobile phone.

### 3.2. Mask R-CNN Method

Mask R-CNN can reduce the impact of pixel misalignment compared to other RCNN methods. The general method maps image features from original image area to convolution area through RoI (Region of Interest) Pooling. This operation makes the coordinates of the convolved image one-Nth of the original coordinates. Therefore, the integer point coordinates in the original feature map appear to be decimal after the calculation and have to be rounded, which leads to the actual ROI area and the extracted features not aligned. To solve this problem, Mask R-CNN replaced ROI Pooling with ROIAlign. The ROIAlign method accurately calculates the pixel information by bilinear interpolation [35], which largely avoids the pixel misalignment caused by the RoI method, thereby ensuring the accuracy of the following distance calculation.

**Figure 4:**
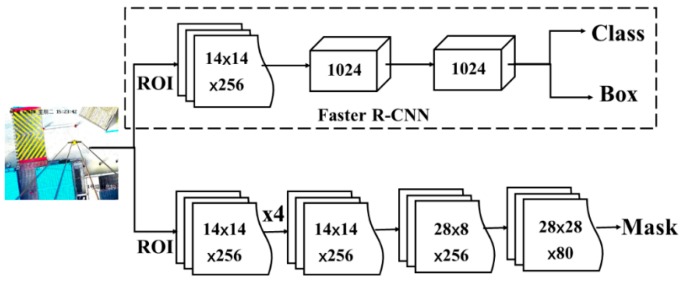
The diagram of the Mask R-CNN identification process.

Because a new mask layer is added to the result, the impact of mask loss should also be considered when calculating the total loss value, as shown in Figure 4.

(1) $${L=L}_{\mathrm{cls}}+L_{\mathrm{box}}+L_{\mathrm{mask}}$$

In which, *L*_cls_ is a classification loss, *L*_box_ is a candidate box regression loss, *L*_mask_ is a mask layer loss.

### 3.3. Safe Distance Calculation

(1) Mask layer extraction

What we have to do is to extract the mask layer of a specific color from the original image. As we know the color image is composed by R (red), G (green), B (blue) channels, and each channel is represented by the number from 0, 1, 2... up to 255. Therefore, this research extracts mask layer by inputting the corresponding RGB values.

(2) Edge extraction

All the pixels can be obtained by step (1). Because direct extraction will increase the calculation time and result in too many pixels to achieve real-time monitoring. Therefore, data reduction is necessary. We compiled a edge extraction program to extract the outermost edge pixels.

(3) Edge coordinate extraction

**Figure 5:**
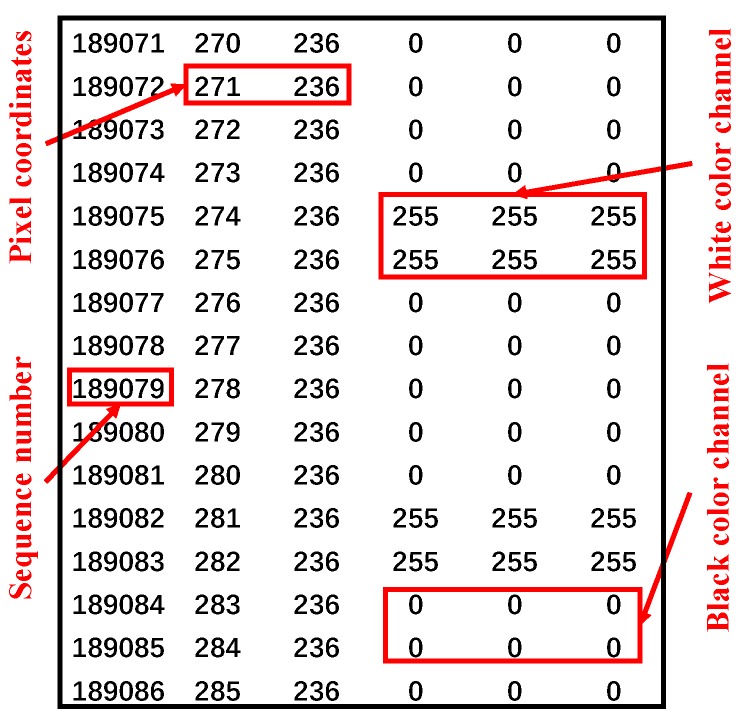
Pixel coordinate extraction diagram.

Since we have unified the resolution of each picture to 800 × 797 in image input process, a total of 637,600 pixels can be extracted as shown in Figure 5. Among them, the numbers of “255, 255, 255” represent the background coordinates and the number of “0, 0, 0” represent the target edge coordinates.

(4) Distance conversion

As pixel distance is proportional to the actual distance, so Equation (2) is known.

(2) $$\frac{L_{1}}{L_{2}}=\frac{A_{1}}{A_{2}}$$

In which, *L*_1_ is the maximum pixel distance of the hazard source, *L*_2_ is the minimum pixel distance between the worker and the hazard source, *A*_1_ is the maximum actual distance of the hazard source, *A*_2_ is the minimum actual distance between the worker and the hazard source.

In this experiment, we have measured the actual length of *A*_1_ on site. In fact, because BIM technology is popular, the size of catchpit can be read by BIM conveniently. In order to get *L*_1_, we calculated the Euclid Distance of all the hazard pixels obtained in step (3) and kept the maximum value. Similarly, to get *L*_2_, we calculated the Euclid Distance between the hazard pixels and the purple worker pixels. After that, the minimum value is retained.

## 4. Experiment

### 4.1. Collection of Test Images

In order to facilitate the driver’s observation, a camera in the vertical state will be installed on the tower crane boom, as shown in Figure 1. The test images of this research are from full-day video recording of the camera on a Dalian construction site. Actually, the research is also suit for any vertical camera at height.

(3) $$H_{4}=H_{1}+H_{2}+5.7+50*sin\alpha$$

In which, *H*_1_ refers to the vertical distance from the embedded end of the tower crane to the ground, measured by *H*_1_ = 138.2 m. *H*_2_ refers to the vertical distance from the embedded end of tower crane to the control room, which includes seven mask section, each mask section size is 1.6 m × 1.6 m × 3 m; *H*_3_ represents the distance between the hook and the camera. α represents the angle between the tower crane boom and horizontal plane.

**Figure 6:**
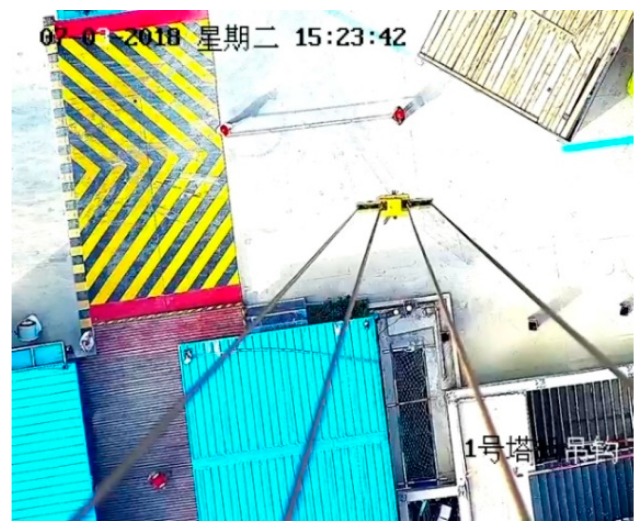
A sample image taken by the tower crane camera at the construction site.

A total of one hour working videos are recorded during three periods of time. After that, we take screenshots at a frequency of 10 s. The 10 s is chosen because the screenshot image would be too repetitive if the time is close. Finally, 600 photos are selected to train model of hazard, workers, and tower cranes hook. The sample picture is shown in Figure 6. At the screenshot time, H3 = 88.3864 m, α = 27.9. According to Equation (3), the camera height *H*_4_ could be estimated to 188.2964 m.

### 4.2. Operating Environment and Parameters Settings

The Mask R-CNN framework was tested using a video to detect workers who were in danger. The framework was performed on a server with Intel Core i7-8700 CPU, NVIDIA 1080ti GPU, 32 RAM. The compiled language of the software was python, and the tensorflow deep learning framework was used to transmit complex data structures to the artificial intelligence neural network for analysis and processing.

The parameters used to train Mask R-CNN were: (1) epoch was equal to 30, each epoch had 20 steps, a total of 600 steps; (2) five anchor scales were used (8 * 6, 16 * 6, 32 * 6, 64 * 6, 128 * 6); (3) the learning rate of the initial 20 steps was 0.02. At the same time, the learning rate was reduced by one tenth for each additional 10 steps; (4) batch size = 2.

**Figure 7:**
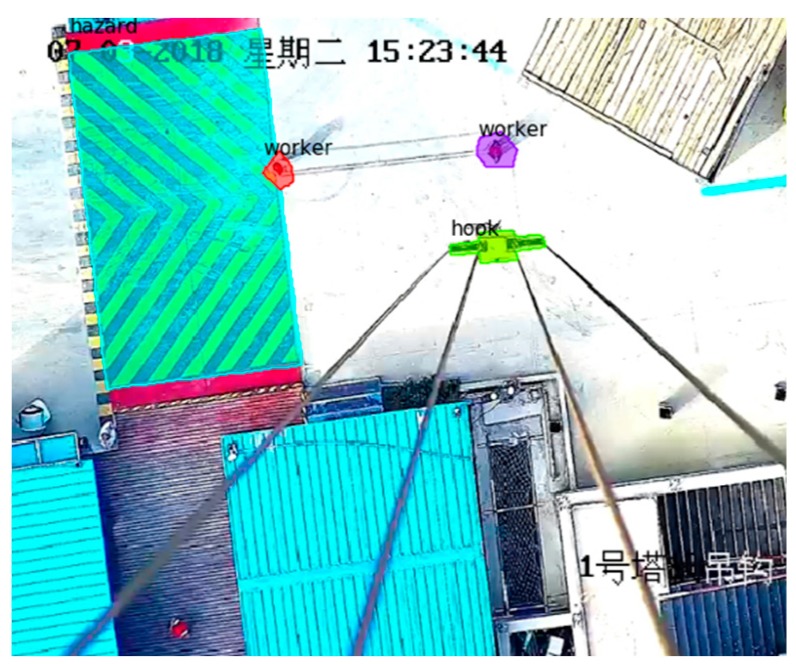
Recognition result of sample image through the Mask R-CNN method.

In the testing phase, Mask R-CNN was characterized by a candidate box network branch and NMS (non-maximum suppression) to predict the bounding box. To be specify, the mask network branch was used to process 100 candidate boxes with the highest scores. Although the mask network branches would predict K (K is the category label for the classification network branch prediction) masks for each RoI, only the Kth mask was used here and the color was assigned. Here is a recognition result in Figure 7.

### 4.3. Results and Discussion

To interpret the recognition performance, four loss values have been calculated as shown in Equation (1). Through the Tensorboard panel, we can get the trend of the four loss values when testing 600 pictures.

**Figure 8:**
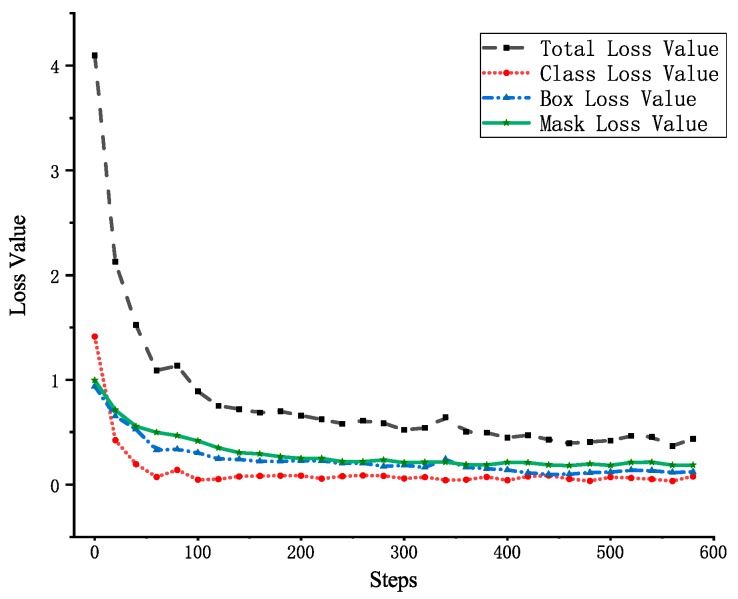
Trend of four loss values during 30 epochs.

It can be seen from Figure 8 that the total loss value can be divided into three phases: (1) The total loss value drops very fast in the first 100 steps; (2) Starting from 100 to 400 steps, the rapid decline becomes a slow decline; (3) The slope tends to be steady at 400 to 600 steps; and finally, the loss value of 600th step is 0.36. In addition, the Box loss value and the Mask loss value have the same trend, but the class loss value is slightly different. In Figure 8, the Class loss value has the highest learning efficiency and the largest decreasing slope, therefore, it begins to stabilize earlier from the 50th step. Above all, four loss values tend to be stable in the last 100 steps. That is to say, more training has little effect on the loss value, so setting it to 600 steps is very reasonable.

In addition, to test the recognition accuracy of the model, 30 images are randomly captured in another video. After testing the 30 images, the recognition results are shown in Table 3.

### 4.4. Safety Distance Conversion

For the workers’ safety consideration, this paper assumes a steel plate with a length of 7255 mm and a width of 4550 mm as the dangerous zone. During the video recording, two workers were asked to hold a measuring scale synchronously, at the same time, two requirements were executed. On one hand, this ruler is kept perpendicular to one side of the source of danger, and on the other hand, it is fixed to a length of 5 m. By this way, it can be considered that ’5 m’ is the target safety distance this paper eventually wants to calculate

**Figure 9:**
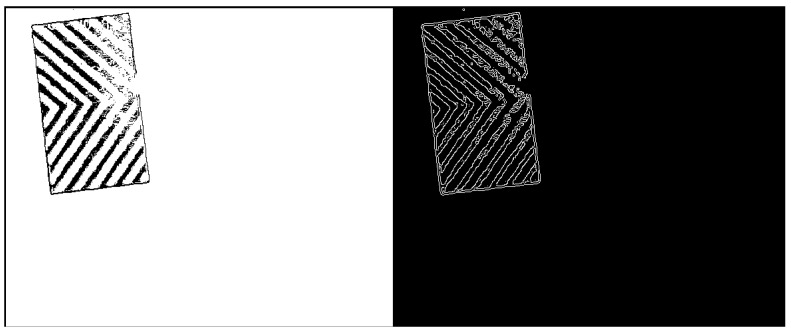
Pixel coordinate extraction process of hazard source mask layer.

After the processing of recognized image by steps 4.4(1), (2), the following two images can be obtained respectively in Figure 9.

Then, pixel coordinates are extracted as Figure 5.

Since we have unified the resolution of each picture to 800 × 797 in the image recognition process, a total of 637,600 pixels are extracted. As shown in Figure 5, for example, the pixel coordinates of line 189,078 is 277 × 236, and the pixel points are black. After eliminating all “0, 0, 0” pixels, it can be calculated that the maximum danger source pixel coordinate distance is 300.6959 pix and the worker’s maximum pixel distance is 39.8 pix. The minimum pixel coordinate distance between the worker and the hazard source is then calculated to be 150 pix. Finally, according to the equivalent conversion, the actual distance between the person and the dangerous source is 4.852 m (actually 5 m) and the error is 3%.

**Figure 10:**
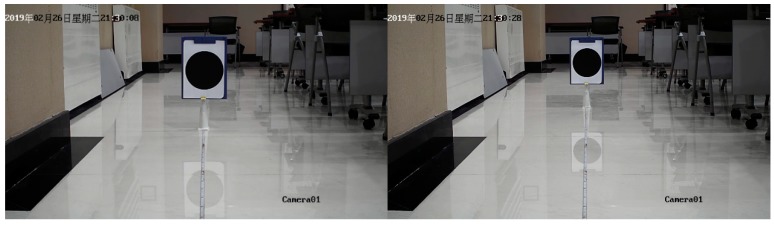
Schematic of the Calibration test.

In order to determine the pixel conversion relationship between different planar objects in the same photo, a set of supplementary experiments had been done, as Figure 10.

**Figure 11:**
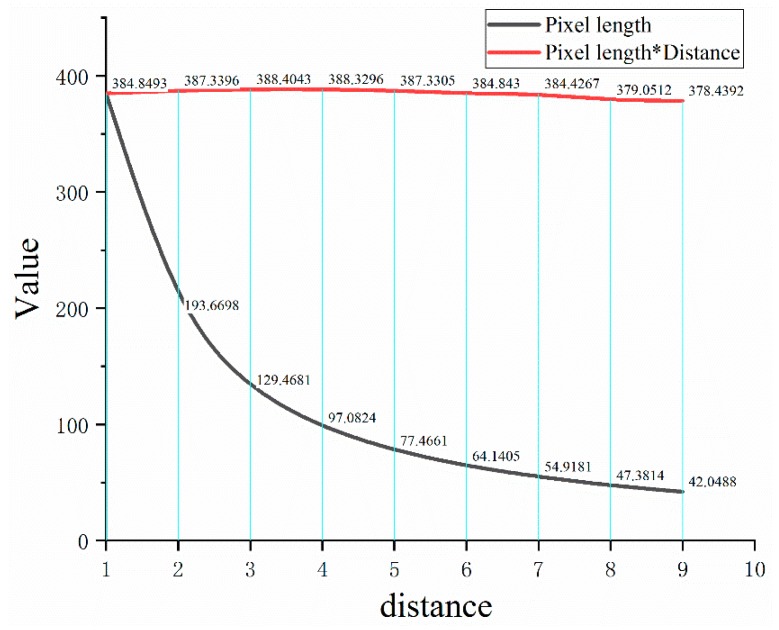
Trend between pixel length and distance.

The video was recorded by using Hikvision camera. There are two requests during the experiment. First, calibration object and camera were always in the same horizontal plane. What is more, the starting position was 1 m away from the camera and shifted backwards at a distance of 1 m each time until 9 m away. Further distance has no reference value because the area of the calibration displayed on the picture is too small. The results are shown in the Figure 11.

As shown by the red line, the product of pixel length and distance remains around 384. Thus, it can be inferred that:

(4) $$\frac{H_{4}\times L_{2}}{A_{2}}=\frac{L_{3}\times H_{3}}{A_{3}}$$

In which, *L*_3_ is the pixel distance of the hook and *A*_3_ is the actual distance of the hook.

During the experiment, it is measured that *A*_3_ = 1.07 m, *L*_3_ = 74.5453 pix. Then, the safety distance *A*_2_ can be converted to 4.587 m by the actual length of the hook, and the error is 0.413 m.

## 5. Discussion

In this paper, the on-site hazard, hook, and workers are identified by Mask R-CNN, and the safety distance is converted by pixel coordinate extraction method. Real-time monitoring of the distance can provide some help to tower crane driver for better work and reduce the occurrence of struck-by accidents. Compared with traditional methods, this research has the following three advantages:

First, it would be a large workload to build and maintain a dense RFID environment. Meanwhile, the computer and camera are now basically configured at the construction site. The application of Mask R-CNN method on the construction site eliminates the need to purchase new hardware facilities and provide a non-invasive method for auxiliary safety construction. Second, allowing a twist angle between the camera and the recognition object. As previous researches limited by the anchor principle, positional information would be out of alignment if the object and the lens have a torsion angle. To solve this problem, we use the Mask R-CNN method to add a mask layer, which can be completely covered on the recognition object. This method also provides guarantee for the conversion accuracy of the subsequent safety distance. Third, small error of the predicted distance. In above experimental part, the distance error obtained by pixel conversion method is 0.15 m. In the subsequent revision process, we find that the occlusion of hazard source by the red workers would affect the recognition effect. Then, a supplementary calculation is implemented to calculate the distance between two workers. After the program calculation, the maximum pixel distance of the red worker is equal to 29.4109 pix and the minimum pixel distance between the red and purple worker mask is equal to 139.1760 pix, that is to say the red and purple worker’s mask center distance is 176.056 pix. Through formula 2, *A*_2_ = 5.025 m is obtained and the error rate is 0.4%. Finally, this research gives the pixel distance conversion relationship between different planar objects. The experiment can help achieve the actual distance of all objects on the image rather than just the same plane. This provides the possibility to implement more functions for camera.

The above lists several advantages, while two limitations still exist in this method.

First, since the image recognition work is limited by the photographic mechanism, only the working plane can be shooted and the indoor situation cannot be as transparent as the WIFI and RFID technologies. Moreover, because it is not feasible to add a surveillance camera to every room, the detection range is limited. Next, the image acquisition angle of this article is fixed. To get the best precision, it needs to be perpendicular to the work surface. If the angle between the ground and the ground is larger, the error of the longitudinal distance would be increased.

## 6. Conclusions

The on-site danger sources are hidden and numerous. While the ordinary worker’s attention is mostly concentrated on his own work and often neglects the surrounding dangers. Therefore ensuring personal safety is the top priority in construction. Lots of research have been done to protect worker from hazard zone. However, price issues, accuracy issues etc. all restrict the practical application of these studies. In this paper, the on-site camera and the Mask R-CNN-based image recognition program are used to identify field workers and hazard sources without adding external hardware devices. A color layer is created on the identified object to provide an environment for subsequent work. At the same time, extracted loss value and AP value are analyzed. The results show that this method satisfies both the recognition accuracy and the reliability. Based on this high-precision recognition method, the pixel coordinates of mask layer can be extracted by adjusting different RGB thresholds. Then, the maximum pixel distance of the hazard source and the minimum distance between worker and hazard source are obtained by cross-iterative method. Finally, this study obtain a high-precision safety distance evaluation model, which can provide assistance for tower crane drivers.

## 7. Future Work

So far, this research has only experimented with camera fixed on tower crane. According to the theory, installing a wide-angle camera on the roof of each room can achieve the same effect. Next, in terms of computational efficiency, the task that consumes the longest time is pixel distance conversion (Table 4.). This is because there are thousands of pixel points generated for each Mask. The current total calculation time is expected to be insufficient to meet the real-time requirements, but better hardware and optimization algorithms can expedite computing efficiency. In addition, this study has an early warning device around the source of danger. Once a worker approaches, it will trigger an alarm, which will also affect other workers around. To meet this problem, we will continue to study how to only notify the individuals who are in danger such as signaling their mobile phones, so as not to interfere with the other normal works.

## Figures and Tables

**Table 1:** List of safety distance statistics in specification.

Events	Safety Distance (m)
Concrete jet operation process	5
Frog type smashing machine operation process	2
Percussion hammer operation	6
Compressed air flushing pipeline process	10
Earth excavation process	2–3
The process of piling foundation hole	3
The process of lifting the asphalt to the operating surface	10

**Table 2:** Statistical zone division.

Zone Division	CL	Zone1	Zone2	Zone3	UCL<
Distance from hazard (m)	13	10–13	7–10	4–7	0–4

In which, CL is statistical Center line, UCL is Upper Control Limit.

**Table 3:** Recognition average precision (AP) for different objects.

Ap for Hazard	Ap for Hazard	Ap for Hazard	Mean Average Precision
0.987	0.985	0.988	0.986

**Table 4:** Time for each step in the proposed framework.

Step	Computation Time (s)
Image identification	0.403
Mask layer extraction	0.102
Edge extraction	0.070
Edge coordinate extraction	0.321
Distance conversion	1.061
Total	1.957
